# From cells to organs: progress and potential in cartilaginous organoids research

**DOI:** 10.1186/s12967-023-04591-9

**Published:** 2023-12-21

**Authors:** Xiao-he Wang, Ning Liu, Hui Zhang, Zong-sheng Yin, Zhen-Gang Zha

**Affiliations:** 1 Department of Bone and Joint Surgery, the First Affliated Hospital, Jinan University, Guangzhou, 510630 Guangdong China; 2 Department of Orthopaedics, The First Affiliated Hospital of Anhui Medical University, Anhui, China

**Keywords:** Organoids, Spheroids, Cartilage, Tissue engineering, Pluripotent stem cells

## Abstract

While cartilage tissue engineering has significantly improved the speed and quality of cartilage regeneration, the underlying metabolic mechanisms are complex, making research in this area lengthy and challenging. In the past decade, organoids have evolved rapidly as valuable research tools. Methods to create these advanced human cell models range from simple tissue culture techniques to complex bioengineering approaches. Cartilaginous organoids in part mimic the microphysiology of human cartilage and fill a gap in high-fidelity cartilage disease models to a certain extent. They hold great promise to elucidate the pathogenic mechanism of a diversity of cartilage diseases and prove crucial in the development of new drugs. This review will focus on the research progress of cartilaginous organoids and propose strategies for cartilaginous organoid construction, study directions, and future perspectives.

## Introduction

Organoids are intricate three-dimensional (3D) structures that replicate the functionality and complexity of organs in an in vitro setting. These structures are derived from adult stem cells (ASCs) or pluripotent stem cells (PSCs), cultured in a 3D environment that facilitates the formation of intricate cell‒cell and cell–matrix interactions [1, 2]. Over the past decade, the field of organoids has emerged as a powerful tool for investigating various aspects of organ development, disease modelling, drug discovery, and regenerative medicine.

**Fig. 1:**
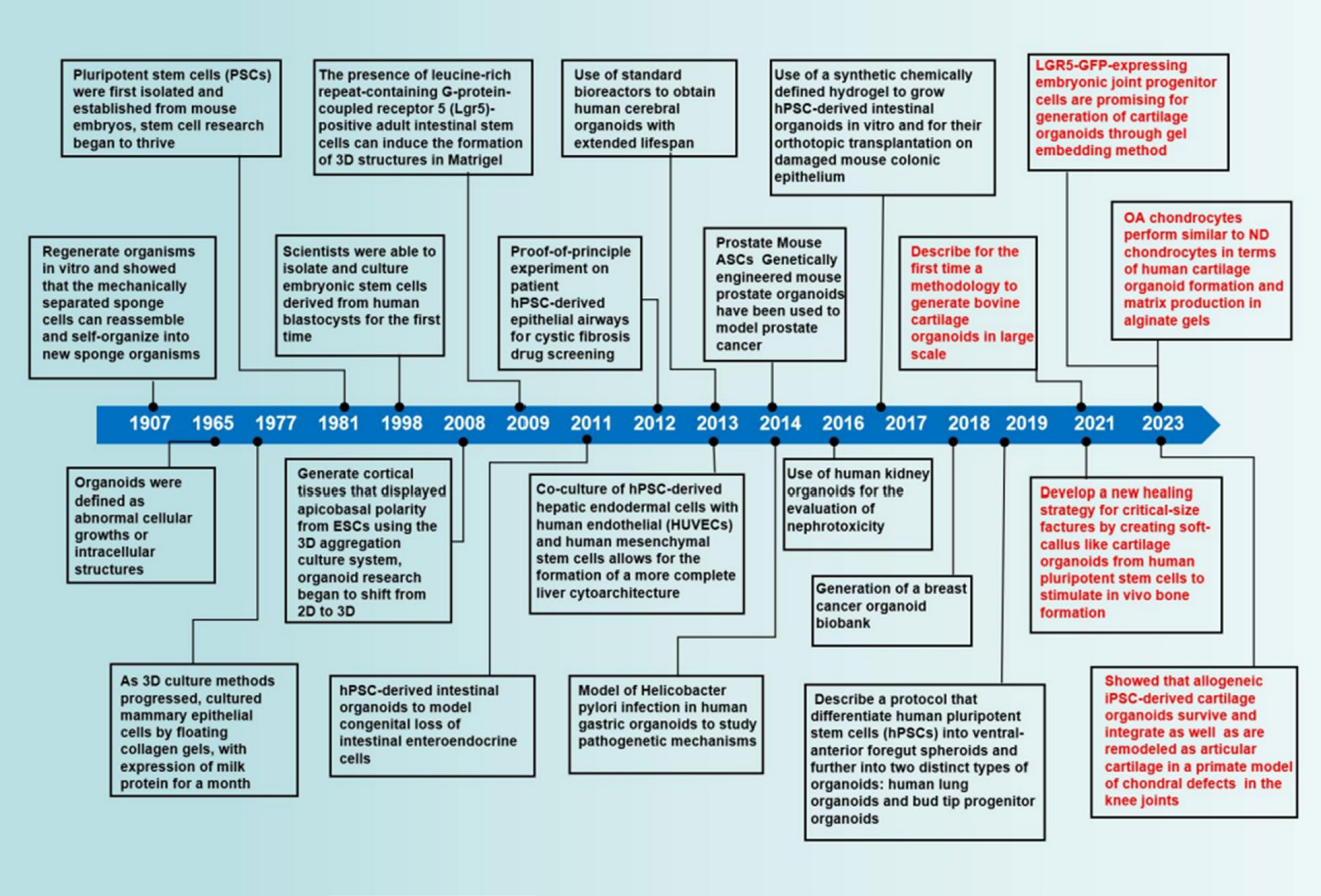
Timeline of milestones for the generation of organoids and the sudden rise of cartilaginous organoids

The concept of organoids has a long history, dating back to the early twentieth century when researchers first attempted to culture tissue explants in vitro [3]. However, the limitations of these cultures in terms of their ability to self-organize and form complex structures led to their abandonment in favor of two-dimensional (2D) cell culture models. It was not until 2009 that Clevers and colleagues developed the first organoid system, demonstrating that a single intestinal stem cell could give rise to a self-organizing, 3D structure that replicated the architecture and function of the intestinal epithelium [4]. Since then, organoids have been generated for a wide range of organs, including the liver, pancreas, lung, kidney, brain, and retina [5–15] (Fig. 1). However, several significant challenges persist in their development and utilization. One pressing issue is the lack of standardized protocols for generating and characterizing organoids [16]. Additionally, the limited availability of specific cell types and the high cost associated with organoid culture present further barriers to the widespread adoption of this technology [17, 18]. As a result, the field of organoid research is still in its early stages, with substantial work remaining to optimize organoid culture systems, enhance their functionality and broaden their applications.

In recent years, the study of organoids has expanded to encompass the realm of cartilage (Fig. 1), providing novel insights into the underlying biology of cartilage and the potential development of therapeutic interventions.

As a novel model of organoids, cartilaginous organoids represent 3D cell clusters that are formed through the differentiation of diverse stem cells possessing self-renewal and self-organization capabilities, utilizing either bioactive materials or not [19]. These organoids have the capacity to mimic the morphology and certain functions of cartilage tissue and can be substantially expanded in vitro.

This review aims to provide an overview of cartilaginous organoids, discussing their recent advances, potential applications, and the challenges that current methodologies must overcome. Furthermore, we will explore future directions for the field and potential advancements that could further enhance the utility of cartilaginous organoids in research, drug development, and personalized medicine.

### Basic structure and pathophysiology of cartilage

Cartilage, a specialized form of connective tissue, is characterized by its low cell density and high matrix composition. The extracellular matrix (ECM) of cartilage consists of a complex arrangement of collagen fibres and proteoglycan molecules, providing the tissue with a unique ability to withstand compression. Chondrocytes, the primary cell population in cartilage, play a crucial role in synthesizing and maintaining the matrix in response to various genetic and environmental cues, including growth factors and physiological loading [20]. However, under pathological or injurious conditions, chondrocytes may transition to a degradative and inflammatory phenotype [21].

**Fig. 2:**
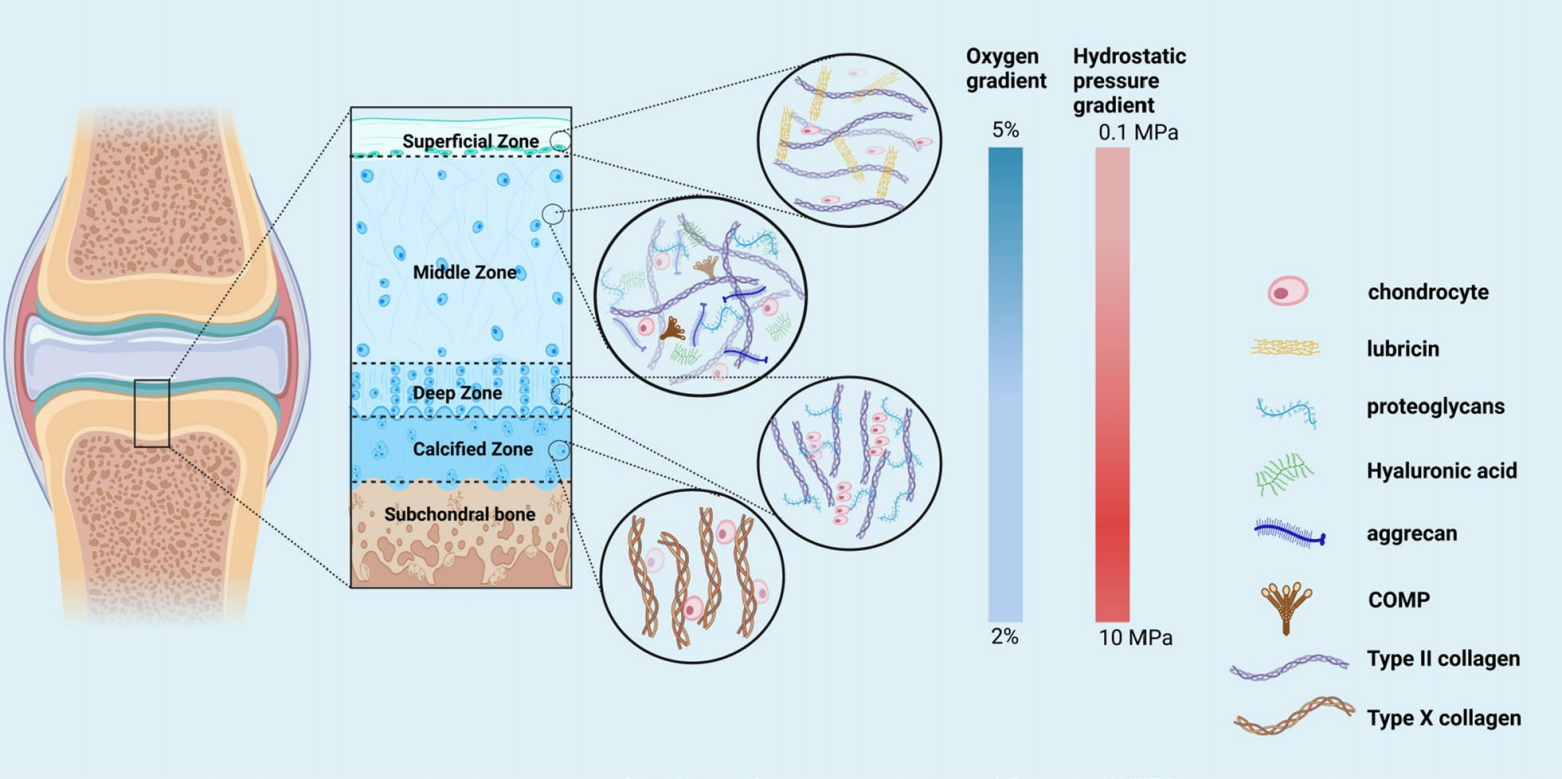
Schematic diagram describing the zones within articular cartilage. Articular cartilage can be divided into four different zones (superficial, mid, deep and calcified), each of which has a characteristic composition and structure and there are changes in oxygen tension and hydrostatic pressure gradient from the superficial zone to the calcified zone

As a vital component of the musculoskeletal system, cartilage possesses distinctive features such as the absence of blood vessels and nerves. It serves as a structural support system and enables smooth joint movement by cushioning bones and reducing friction between them [22]. Articular cartilage, specifically, is anatomically and functionally divided into four distinct zones, characterized by distinct morphological, chemical, and collagen density properties [23] (Fig. 2). Despite its critical role, cartilage has limited self-repair capacity, rendering it vulnerable to various pathological changes and diseases such as osteoarthritis (OA), chondromalacia patellae, and chondral injuries [24].

OA, a degenerative joint disease, is characterized by the progressive loss of articular cartilage. With a growing prevalence affecting more than 30 million Americans, the incidence of OA is escalating due to the aging population and increasing obesity rates [25]. While the exact mechanisms underlying OA remain incompletely understood, it is considered a complex interplay of mechanical, biochemical, and cellular factors [26, 27].

### Existing cartilaginous preclinical models

**Fig. 3:**
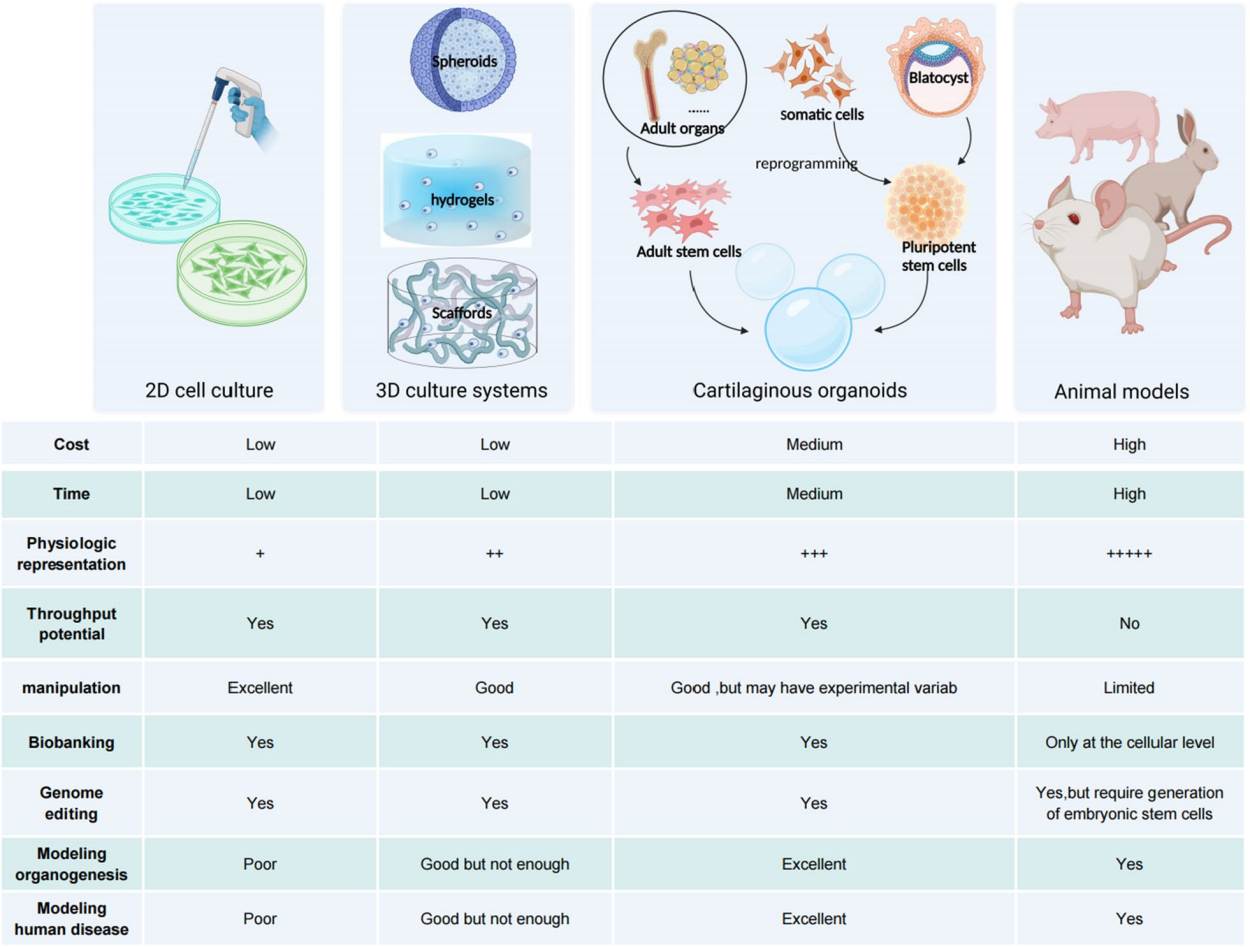
Comparison of cartilaginous organoids with other preclinical models. Cartilaginous organoids can be generated from stem cells in adult tissue or from pluripotent stem cells. In serving as a bridge between conventional two-dimensional culture and animal models, cartilaginous organoids retain the merit of both and put right their deficiency to some extent

Preclinical models play a crucial role in the advancement of treatments for cartilage diseases by enabling the investigation of underlying mechanisms, testing the effectiveness and safety of new therapies, and predicting their clinical potential (Fig. 3).

#### In vitro models

In vitro models are cell-based systems that provide a controlled environment for studying the biological behavior of chondrocytes. Primary cell cultures, immortalized cell lines, and engineered cartilage constructs are commonly used in vitro models for cartilage research.

Primary cell cultures involve the use of fresh cartilage tissue to study chondrocyte behavior. However, their use is limited by the availability of fresh tissue, the short lifespan of chondrocytes in vitro, and challenges in maintaining their phenotype [28].

Immortalized cell lines like ATDC5 and C28/I2, derived from chondrocytes, offer a continuous source of cells for research purposes. However, these cell lines exhibit distinct biological properties compared to primary chondrocytes, limiting their ability to accurately model normal cartilage behavior [29, 30]. However, these cell lines have shown different biological properties compared to primary chondrocytes, which limits their usefulness in modeling the behavior of normal cartilage cells.

To overcome the limitations of 2D cell expansion, researchers have explored various 3D culture systems such as pellets, spheroids, and microtissues. These systems enhance chondrocyte viability and chondrogenic potential. The resulting constructs can be encapsulated in hydrogels or scaffolds to generate cartilage with improved quantity and quality compared to 2D systems [31–35]. The transition from 2 to 3D cultures holds promise for developing more physiologically relevant in vitro models of human development and disease. However, current technology still faces challenges in replicating the mechanical and biological complexity of native cartilage and achieving high cell numbers.

#### Ex vivo models

Ex vivo models involve the study of isolated cartilage explants or organ cultures, providing a more physiologically relevant environment compared to in vitro models. Explant cultures [36], where small pieces of cartilage tissue are cultivated in vitro, offer a closer representation of the joint environment. However, these models face constraints due to the limited availability of fresh tissue, the complex and variable nature of the joint environment, and the challenges in maintaining cell phenotype.

#### In vivo models

In vivo models involve studying cartilage and joint function in living animals. These models provide insights into the behavior of cartilage in a physiological environment and are valuable for studying interactions between cartilage and other joint tissues. The most commonly used in vivo models for cartilage research are animal models.

Animal models, such as mice and rabbits [37], have been extensively used to study the mechanisms underlying cartilage degeneration and test the efficacy of new therapeutic strategies. However, these models have limited translational potential due to anatomical and biological differences between animals and humans, which can compromise result validity.

The pharmaceutical industry is currently examining the reliability of in vitro assays conducted during the preclinical phase of drug discovery, particularly those utilizing 2D cell cultures and animal models. Criticisms have been raised regarding the limited physiological similarity of these models to healthy or diseased human tissues [38], while animal models are also criticized for their prolonged testing period, high expenses, and ethical concerns.

The prevailing consensus within the scientific literature is that advanced 3D cell culture models, derived from human cells, have significant potential for enhancing drug development predictions [39]. The advent of human adult stem cells, including mesenchymal cells, and human induced pluripotent stem cells (iPSCs) has made it possible to create intricate 3D models. These cells can accurately mimic the morphogenetic events of tissue and organ development, and their intrinsic differentiation capacity is maximized in 3D cell culture models utilizing non-adherent surfaces or matrigel. These models are commonly referred to as organoids [40].

### Progress in cartilaginous organoids

#### Starting cell type of cartilaginous organoids

Over the past two decades, stem cell research has significantly expanded our understanding of critical aspects of cartilage organogenesis by harnessing the self-organizing properties of PSCs and ASCs. These self-organizing properties refer to the ability of cells within an organoid to arrange themselves into a structure that closely resembles the original tissue, facilitated by specific signaling pathways. In this context, spheroids derived from ASCs can be considered organoids because they effectively replicate tissue morphogenesis and mimic at least one tissue/organ function [41].

#### PSCs

PSCs, including embryonic stem cells (ESCs) and induced pluripotent stem cells (iPSCs), possess the remarkable capability to differentiate into virtually any cell type. In recent years, iPSCs have garnered significant attention due to their potential applications in regenerative medicine and disease modelling. Of particular interest is their use in generating cartilaginous organoids [42].

**Fig. 4:**
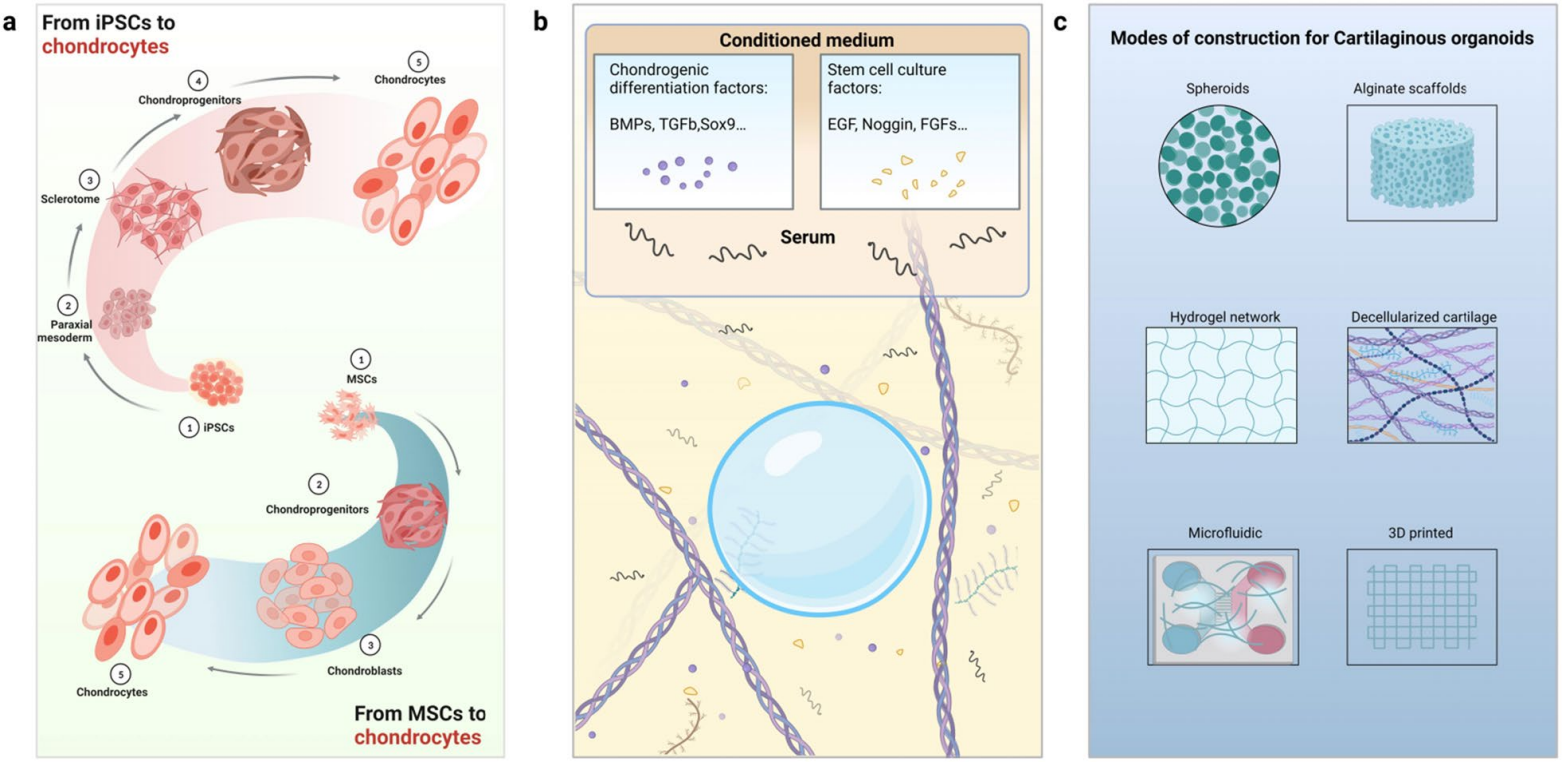
Current understanding and progress of cartilaginous organoids. **a** Overview schematic of the protocol of chondrogenic differentiation from iPSCs and MSCs; **b** The use of ill-defined and heterogeneous medium components for organoid cultures, including conditioned medium and animal-derived serum, which unpredictably alter organoid phenotype; **c** Various methods and scaffolds have been used for the construction of cartilaginous organoids. These scaffolds have a complex and ill-defined composition as well as overall poor tunability, which limits the studies of cartilaginous organoids. In summary, protocols used throughout cartilaginous organoid derivation and culture are nonstandardized, which is detrimental to the reproducibility of cartilaginous organoids

Insights from developmental biology have revealed the sequence of inductive and repressive signaling pathways necessary for PSC lineage specification to different cell fates [43, 44]. Robust and stepwise differentiation protocols have been reported to drive iPSCs towards a chondrogenic lineage via the paraxial mesoderm [45, 46] (Fig. 4a), positioning iPSCs as a promising cell source for cartilage tissue engineering [47].

In recent years, iPSCs have emerged as a versatile tool for investigating cartilaginous organoid formation [48–50]. The development of iPSC-based cartilage regeneration therapies is a rapidly evolving field with exciting potential applications. iPSCs have shown particular effectiveness in modelling monogenic cartilage diseases, and recent studies have produced positive results in the modelling of skeletal dysplasia [51].

iPSCs share many similarities with ESCs, including unlimited self-renewal and pluripotency, surface marker expression, and telomerase activity [52–54]. Although patient-specific ESCs can be generated through somatic cell nuclear transfer (SCNT), the widespread application of SCNT to human cells is limited by the ethical concerns surrounding the use of human embryos in research. In contrast, iPSCs offer a promising alternative cell source for cartilaginous organoids as they do not require the destruction of human embryos, thereby bypassing ethical and political issues associated with the use of ESCs [53].

Despite their advantages, challenges persist in generating functional cartilaginous organoids from iPSCs, such as producing them in large quantities and maintaining their functionality over time. The potential for cells derived from iPSCs to dedifferentiate or become tumorigenic also poses a significant hurdle for clinical applications [55, 56]. Furthermore, iPSC heterogeneity is influenced by donor mutations, epigenetic memory of reprogrammed cells, and the iPSC production system employed [17, 57].

Therefore, further research is needed to address these challenges and improve the reproducible differentiation of iPSCs into chondroprogenitors (CPs) for cartilage tissue engineering and modelling of arthritis.

#### MSCs

Mesenchymal stem cells (MSCs) have emerged as promising candidates for regenerative medicine due to their capacity to differentiate into various cell types, including chondrocytes. In the clinical setting, subchondral drilling, a marrow stimulation technique, is widely employed for articular cartilage repair and relies on the chondrogenic differentiation potential of MSCs [58].

The chondrogenic differentiation of undifferentiated MSCs encompasses several processes, including proliferation, maturation, and terminal differentiation [59]. Notably, it is possible to produce cartilaginous organoids by culturing MSCs in vitro using specific growth factors and extracellular matrix molecules [60]. The advantages of utilizing MSCs for the development of cartilaginous organoids lie in their accessibility, rapid expansion capability, and ability to differentiate into cells from various mesenchyme-derived tissues [61, 62]. Among these, cells derived from bone marrow, synovium, and periosteum have shown the highest potential for chondrogenesis [63, 64] (Fig. 4a).

Adipose stem cells and bone marrow MSCs have been extensively studied in cartilage tissue engineering due to their similar biological characteristics [65, 66]. However, it has been reported that bone marrow MSCs demonstrate a higher capacity for osteogenic differentiation than adipogenic stem cells [67].

Although the utilization of MSCs in cell-based tissue engineering and regenerative medicine holds promise, there are challenges to address. These include the variability in MSC properties across different sources and the difficulty in creating organoids that closely resemble native cartilage [68]. These inconsistencies may arise from the inherent heterogeneity associated with MSC populations in terms of cell proliferation capacity and differentiation potential, which can pose significant challenges for their application in tissue engineering [69, 70].

Several studies provide further evidence supporting the long-term chondrogenic potential of iPSCs compared to MSCs. iPSCs tend to exhibit higher expression levels of collagen types I and X and undergo hypertrophy relatively quickly [48, 71]. However, it is premature to conclude that iPSCs are superior to MSCs for the formation of cartilaginous organoids. Nevertheless, iPSCs can address several issues, such as cell number, fibrocartilage formation, or phenotype loss with passages [72].

Moreover, human periosteum-derived cells (PDCs) share similar characteristics with MSCs and can be used to produce chondrocyte microspheres that differentiate into callus organoids [73]. Additionally, Lin et al. suggested that LGR5-GFP-expressing embryonic joint progenitor cells hold promise for generating cartilage organoids through the gel embedding method [74].

### Construction of cartilaginous organoids

During various developmental processes, the gradients of morphogens and physical cues play a critical role in determining the polarity and diversity of structures that form in vivo. Similarly, self-organizing organoid systems have the ability to establish complex cellular patterns through successive modifications of the local microenvironment, driving organoid morphogenesis in vitro.

#### Physical characteristics of the cultural environment

Currently, numerous tissue engineering techniques are being developed to form cartilaginous organoids. One significant distinction among these approaches is whether they are scaffold-based or scaffold-free [75, 76] (Fig. 4c).

The scaffold-free culture method offers several advantages over its counterpart, particularly in therapeutic and high-throughput drug screening applications, due to its simplicity and reproducibility [77]. Several studies have utilized human pluripotent stem cell (hPSC) mesoderm to induce chondrocyte differentiation, which then self-assembles into cartilaginous organoids that have demonstrated efficacy in repairing critical-sized bone defects in mice [49, 78]. Scaffold-free tissue engineering primarily relies on multicellular spheroids as the fundamental building block owing to their ease of handling.

While the scaffold-free approach holds theoretical benefits for mimicking natural tissue morphology, combining cartilaginous organoids with suitable biomaterials can further enhance organoid generation and improve their performance. The scaffold can take various forms, such as a classic 3D construct with interconnected pores, a Matrigel or hydrogel with embedded cells, or a combination of both [79].

Solid ECMs are commonly used to promote the 3D characteristics of organoids, providing structural support to maintain cell identity and function [80]. In terms of chondrogenic differentiation of MSCs, Allen et al. found that the appropriate stiffness of the ECM had a synergistic effect on exogenous growth factor stimulation, promoting chondrogenesis [81]. Matrigel, a natural ECM purified from Engelbreth-Holm-Swarm mouse sarcoma [82], is the most widely used matrix for 3D organoid derivation. Its complex mix of ECM components and growth factors makes cell growth and differentiation highly efficient. However, the heterogeneous composition of Matrigel makes it difficult to manipulate the matrix to facilitate various morphogenetic processes [83]. To address this issue, chemically defined hydrogels have been introduced as substitutes for natural matrices to support cartilaginous organoid culture. The impact of hydrogel properties, such as elasticity, swelling, and fixed charge, on chondrogenic behavior has also been reported [84, 85].

For instance, Crispim et al. [86] found that elastic hydrogels constrained the growth and fusion of organoids, inhibiting tissue formation, whereas viscoelastic hydrogels allowed for the growth and fusion of organoids into homogeneous tissue rich in collagen type II and glycosaminoglycans. Xiahou et al. developed a smart and responsive hydrogel that utilizes disulfide bonds as a cellular response switch to create cartilage microtissues in vitro. This hydrogel allows for cell attachment, detachment, and the automatic formation of stem cell aggregates without the need for artificial stimuli, resulting in the formation of cartilage microtissues [87].

Similarly, solid scaffolds can also be derived from natural and synthetic materials, and they must recreate an extracellular matrix in which cells in spheroids can adhere, proliferate, and differentiate [88]. Many studies have investigated innovative approaches for integrating chondrocytes into scaffolds, such as the use of culture in alginate beads or scaffolds, which induce chondrogenic differentiation of MSCs in vitro [89].

In the context of chondrogenesis, the utilization of spatial cues through scaffold design has been extensively studied [90]. Previous investigations have demonstrated that modifications in scaffold density reduction, as well as gradients in pore size, can promote chondrogenic induction in scaffolds, influencing the differentiation of MSCs into chondrocytes [91].

More recently, Yang et al. employed a microfluidic technique to fabricate spatially controlled scaffolds with a highly ordered and uniform porous structure [92]. Rabbit ADSCs were seeded onto these scaffolds to assess the regulation of spatial cues on chondrogenesis. These investigations suggested that the geometry of the scaffold significantly impacted the chondrogenic differentiation of ADSCs, highlighting the critical importance of scaffold dimensionality and geometry in modulating the chondrogenic differentiation of stem cells.

Natural solid scaffolds, such as collagen scaffolds and decellularized cartilage, have been widely utilized in tissue engineering. Decellularization is particularly significant because it preserves the bioactive signals present in native cartilage, which guide cellular events such as adhesion, proliferation, and differentiation. Utomo et al. demonstrated the potential of decellularized ear cartilage scaffolds in vitro, while Kang et al. reported full-thickness repair in a rabbit femur model using adipose stem cell-loaded decellularized cartilage extracellular matrix scaffolds [93, 94]. However, an important drawback of using autologous or allogeneic decellularized cartilage is the potential for donor site morbidity [95].

Traditional tissue engineering scaffolds typically allow for cell attachment only on the surface, with limited control over cell distribution and migration within the scaffold, potentially leading to suboptimal clinical outcomes [83, 96].

Nevertheless, the field of bioprinting has made significant strides toward the generation of complex and fine bionic tissue constructs [97–99]. During bioprinting, bioinks, mostly hydrogels-carrying cells, are continuously extruded from a bioprinter to model functional tissue systems according to a 'bottom-up' strategy. In one study, an 'ALL-IN-ONE' bioink based on granular hydrogel was fabricated, possessing multiple functions such as effectively producing ASC spheroids, volume shrinkage and swelling to combine chondrocytes, and direct extrusion 3D printing for further coculturing ASC spheroids and chondrocytes to incubate chondroids, ultimately showing similar histological characteristics to cartilage tissue [100].

Scaffold-based tissue engineering represents a promising alternative approach to joint repair. It is worth noting that a study demonstrated the successful fabrication of completely scaffold-free, self-sustainable cartilage constructs by collecting MSCs using a well-defined differentiation protocol and combining bio3D printers with Kenzan needle array technology. This approach may facilitate the resurfacing of larger chondral defects and the creation of a new generation of cartilaginous organoids [101].

#### Signalling factors required for cartilaginous organoid formation

Organoids are commonly generated by exposing cells to specific morphogens at precise time points, leading to the activation of desired developmental pathways and subsequent self-organization [2].

Several protocols for chondrogenic differentiation exist, each utilizing different growth factors, intermediate steps, culture times, and systems. However, a consensus has not yet been reached on the most effective approach for chondrocyte generation. Therefore, systematic comparisons of different methods are necessary (Fig. 4b).

Chondrogenic differentiation is regulated by multiple signal transduction pathways that control the condensation of mesenchymal progenitor cells, nodule formation, and subsequent chondrogenic differentiation [99]. The basal chondrogenic medium is supplemented with critical signaling molecules such as BMPs, FGFs, TGFb, Wnt, and cell adhesion molecules (N-CAM, N-cadherin, β-catenin), which have been shown to induce chondrogenic differentiation of PSCs and MSCs [102–104]. These factors activate essential targets that initiate and maintain the chondrocyte phenotype. Furthermore, Sox9, a cartilage-specific transcription factor, is required for mesenchymal progenitor cell condensation and the maintenance of the chondroprogenitor phenotype [99]. Additionally, macromolecules of the cartilage extracellular matrix, such as type II collagen, hyaluronan, aggrecan, or fibronectin, may also serve as signaling molecules [103].

The protocol established by Oldershaw et al. [105] has had a significant impact on stem cell and cartilage research. However, its application to iPSCs has been hindered by low cell viability. Umeda et al. [43] successfully optimized this directed differentiation method by modulating Wnt and TGFb signaling, resulting in improved cell viability and the generation of high-quality hyaline cartilage-like tissue. The identification of key molecules triggering iPSC chondrogenesis in this protocol has made it one of the most impactful approaches in the field.

The protocol by Yamashita et al. [78] involves the initial differentiation of hiPSCs into mesendodermal cells, followed by culturing in a chondrogenic medium supplemented with ascorbic acid, BMP2, TGFb1, and GDF5. The chondrogenically committed cells are then sorted based on collagen type II expression and cultured in 3D, resulting in cartilaginous particles containing rounded cells embedded in an extracellular matrix rich in collagen type II.

Borestrom et al. [106] achieved high-quality chondrogenesis using a protocol that includes a 3D pellet predifferentiation stage, followed by monolayer expansion of chondrogenic progenitors. These progenitors are then cultured in a second chondrogenic 3D pellet and differentiated into chondrocytes using a chondrogenic medium supplemented with growth factors. The gene expression levels of Sox9, type II collagen, aggrecan, and type X collagen are similar to those of human articular chondrocytes.

Chia-Lung Wu and colleagues [107]employed a multiomics approach, integrating bulk RNA sequencing, single-cell RNA sequencing, and weighted gene coexpression analysis (WGCNA) to study the gene regulatory networks that govern hiPSC differentiation into chondrocytes. They identified key hub genes, including WNTs and MITF, and demonstrated that off-target WNT signaling induces chondrocyte hypertrophy in a heterocellular signaling model. Building on this knowledge, Amanda R Dicks and colleagues used small molecules to inhibit Wnt and MITF signaling during chondrogenic pellet culture, significantly improving the efficiency and homogeneity of hiPSC chondrogenesis [45].

By advancing our understanding of the molecular mechanisms underlying organoid formation and function using state-of-the-art technologies and addressing remaining challenges, significant progress in this field is expected in the near future.

### Current application of cartilaginous organoids

**Fig. 5:**
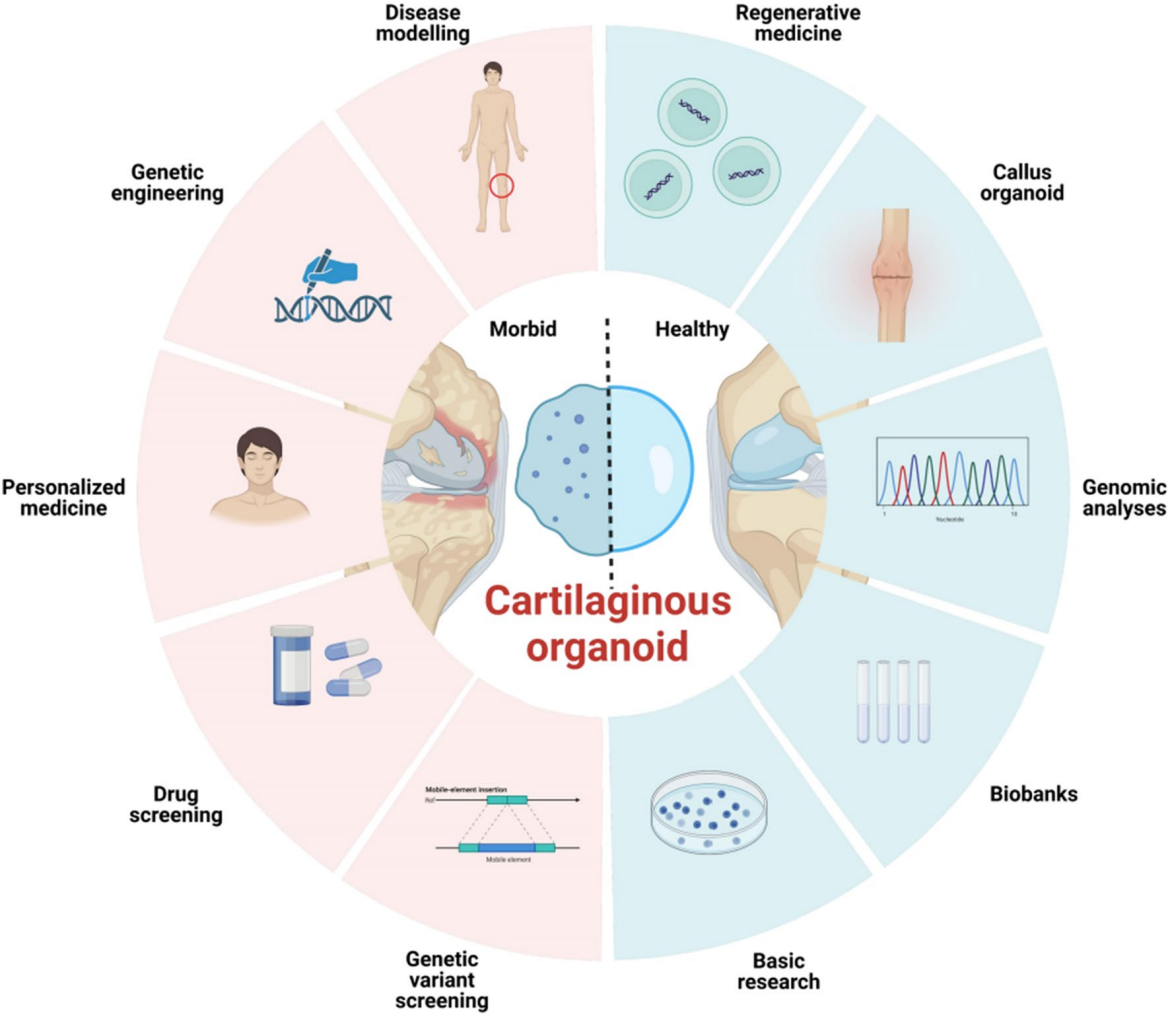
Schematic depiction of applications of cartilaginous organoids. Organoid technology has provided a potential tool for high-throughput drug discovery and enables accurate toxicity testing and preclinical studies in cartilage. By using gene editing techniques such as CRISPR/Cas9, researchers could mimick human cartilage genetic diseases with the help of cartilaginous organoids and further investigate the underlying mechanism. Recent advances in cancer research within cartilaginous organoids are paving the way for promising organoid transplantation therapy in the future

Currently, the utilization of cartilaginous organoids remains somewhat restricted. However, as research progresses, it is foreseeable that the utilization of cartilaginous organoids will flourish in the upcoming decade (Fig. 5).

One of the paramount applications of cartilaginous organoids resides in the realm of disease modelling. These organoids can be derived from patients afflicted with joint ailments such as OA and rheumatoid arthritis, thereby providing researchers with a more physiologically relevant system to investigate the disease. In a recent study, Cullier et al. [108] constructed an equine organoid model for OA by inducing it with IL-1β. The researchers discovered that a combination of BQ-123-CHI and R-954-HA (BR5) yielded the most notable reduction in inflammatory and catabolic markers. This finding underscores the potential of organoids as a potent tool for studying the pathophysiology of joint diseases and developing innovative therapies to combat them.

Another potential application of cartilaginous organoids lies in drug screening. In comparison to conventional animal models, organoids provide a more precise and cost-effective means of assessing the efficacy and toxicity of new drug candidates. This capability not only accelerates the drug development process but also diminishes the necessity for animal testing.

Within the realm of tissue engineering, cartilaginous organoids can be harnessed to pioneer innovative approaches for repairing damaged cartilage tissue. Presently, the existing methods rely on synthetic scaffolds that are seeded with or without chondrocytes or stem cells [109–112]. However, these approaches frequently yield tissue that lacks the functional and mechanical properties of native cartilage. In contrast, cartilaginous organoids present a more intricate and dynamic system for advancing tissue engineering endeavors. Recent studies have underscored the potential of cartilaginous organoids in the realm of tissue repair. For instance, Kengo Abe et al. [113] demonstrated that allogeneic iPSC-derived cartilaginous organoids exhibit remarkable survival and integration capacities, as well as the ability to remodel articular cartilage, when tested in a primate model with chondral defects in the knee joints. Furthermore, Hall et al. [50] ingeniously incorporated genetically distinct populations of cartilaginous tissue intermediates into a single implant, creating an osteochondral tissue unit. By combining human iPSC-derived cartilage microtissues with callus organoids derived from human PDCs, they successfully formed a dual structure of cartilage and bone after implantation. These studies demonstrate the immense potential of cartilaginous organoids as a promising alternative for the repair of damaged cartilage tissue.

Furthermore, cartilaginous organoids present a valuable platform for investigating the long-term behavior of cells and tissues in vitro, which is essential for the development of effective and safe clinical therapies. In pursuit of this objective, Thorup et al. [114] devised an ectopic cartilage formation assay utilizing organoids derived from articular chondrocytes of human donors, which were injected into nude mice to evaluate the potential of bioactive molecules in promoting in vivo cartilage formation. Such test systems based on organoids offer a potent tool for screening molecules with regenerative potential for cartilage.

Moreover, cartilaginous organoids hold significant promise in the advancement of individualized treatments for cartilage-related ailments. By generating organoids from a patient's own cells, therapies can be tailored to the specific biology of each patient, thereby enhancing the likelihood of successful outcomes while mitigating the risk of adverse events. Notably, recent breakthroughs have led to the development of "mini-joint" models that incorporate multicellular components and extracellular matrices of joint cartilage. These models present a novel approach for devising strategies to modify diseases and crafting personalized therapeutics for conditions associated with cartilage [115, 116].

### Limitations and perspectives

Organoids have emerged as potent instruments in fundamental research and have made significant contributions to advancements in the biomedical domain. Nonetheless, the utilization of cartilaginous organoids in translational studies remains restricted due to the intricate and demanding process involved in their translation into practical applications, and achieving maturation and functionality in cartilaginous organoids, remains a significant challenge. This challenge stems from the limited understanding of the molecular mechanisms governing organ development.

### Make progress together with scRNA-seq

Recent studies have demonstrated the remarkable capability of single-cell sequencing (scRNA-seq) in elucidating the cellular heterogeneity and lineage specification of chondrocytes derived from hiPSCs [107], identifying rare cell populations within osteoarthritic cartilage [117], and unveiling the molecular signatures of ferroptotic chondrocyte clusters in human OA cartilage [118]. These investigations underscore the emerging role of single-cell "omics" approaches in chondrocyte research, offering potential insights into innovative therapeutic strategies for OA and other cartilage-related disorders.

The combination of organoid technology and scRNA-seq holds immense potential in addressing the limitations associated with each approach. Significant advancements in stem cell biology have facilitated the precise regulation of differentiation pathways within organoids [119, 120]. Through the integration of cutting-edge technologies for organoid culture and scRNA-seq, researchers have acquired a powerful tool for exploring organ development and diseases [121]. Although the application of these techniques to cartilaginous organoids is still in its early stages, the future appears promising for unlocking their complete potential.

#### Discovery of rare/novel cell types and gene markers

The integration of organoid technology, scRNA-seq, and the increasing availability of organ-specific datasets and machine learning algorithms has facilitated the accurate prediction of cell type identity in organoids. Despite the seemingly simplistic composition of cartilage tissue, primarily consisting of chondrocytes within the extracellular matrix, the tissue exhibits a zonal organization that distinguishes distinct cellular identities. Previous studies have examined the presence of adult cartilage stem and progenitor-like cells; however, their precise identity and characteristics remain subject to debate. By utilizing putative markers for cartilage progenitor cells (CPCs), such as Sox9 and CD44, in addition to CD105/CD90/Notch/Stro/CD151, distinct subsets of CPCs have been identified [117], thereby highlighting the existence of multiple CPC populations. Future investigations should aim to isolate and investigate the specific functions of each of these subsets, particularly their roles in cartilage regeneration and repair.

#### Recapitulation of chondrocyte heterogeneity

A comprehensive understanding of cellular heterogeneity is essential for unravelling the developmental processes and disease pathogenesis of organs. The identification and functional characterization of stem cells and their diverse lineages pose significant challenges in the field of developmental biology [122]. Conventional 2D in vitro models lack the capacity to replicate organ-level cell interactions, underscoring the necessity of 3D organoids for exploring cellular heterogeneity [123]. Progress in organoid and scRNA-seq technologies has facilitated the investigation of chondrocyte heterogeneity in organs. Importantly, the distinct proportions of chondrocyte subtypes may reflect varying degrees of degeneration and immune/metabolic profiles in patients with OA. Therefore, obtaining a single-cell understanding of cartilage can offer higher resolution and innovative insights into the onset and progression of OA pathology.

#### Reveals cell development trajectories during chondrogenic differentiation

The scRNA-seq technique empowers the identification of distinct cell types within an organ at a single-cell resolution, enabling the inference of intermediate cell types and differentiation pathways. In a study conducted by Adkar et al., human iPSCs underwent a chondrogenic differentiation protocol, and scRNA-seq was performed at multiple time points to elucidate the dynamics of molecular signaling pathways and tissue-specific transcription factors during the differentiation process [44]. Notably, Czerniecki et al. discovered that the addition of vascular endothelial growth factor (VEGF) increased CD31 expression in endothelial cells and cadherin expression in vascular endothelial cells within kidney organoids [124]. Hence, the combination of organoid technology and scRNA-seq presents significant potential for identifying chemical compounds that can guide organoid differentiation pathways and facilitate the generation of more complex organoids with enhanced functionality [124].

#### Identification of gene expression variability at the single-cell level

Single-cell gene expression analysis is a powerful tool for elucidating tissue heterogeneity and developmental processes [123]. In a groundbreaking study, Kengo Abe et al. [113] employed a unique approach by combining cartilaginous organoids with scRNA-seq. The researchers induced the differentiation of cynomolgus monkey induced pluripotent stem cells (cyiPSCs) into chondrocytes, resulting in the generation of cyiPSC-derived cartilaginous organoids (cyiPS-Cart). Subsequently, these organoids were allogeneically transplanted into chondral defects on the knee joint surface of cynomolgus monkeys. By utilizing scRNA-seq and conducting molecular analysis of the cyiPS-Cart graft, the study identified molecular pathways associated with cell differentiation, thus revealing gene expression variability. These investigations exemplify how the integration of scRNA-seq and cartilaginous organoids represents a novel approach for understanding the variabilities in gene expression at the single-cell level, thereby enhancing our comprehension of organ development and disease states.

#### Modelling diseases to map cellular heterogeneity in healthy and diseased cartilage tissues

The identification of distinct cell subpopulations and their specific roles in disease states is crucial for precision medicine. In addition to organoids derived from healthy adult stem cells or pluripotent stem cells, those derived from patient-specific stem cells accurately reflect the underlying biology of specific diseases. Moreover, single-cell proteomic analysis allowed the stratification of OA patients into three groups based on the relative proportions of inflammatory and regenerative cells: increased in OA, unchanged between OA and normal, and decreased in OA [117].

The integration of scRNA-seq and cartilaginous organoids presents immense potential for advancing our understanding of cartilage development and the pathogenesis of diseases. However, to fully utilize its capabilities, certain challenges such as incomplete differentiation and limited sensitivity need to be addressed [124]. As datasets are consolidated and new therapeutic targets are identified, the future appears promising. However, the subsequent challenge lies in the development of effective treatments once these targets are recognized.

### Make the most of the epoch-making scissors——gene editing

Among the various gene-editing techniques available, CRISPR/Cas9 stands out due to its precise targeting and shearing capabilities, cost-effectiveness, and user-friendliness, making it indispensable in biological research [125, 126].

CRISPR/Cas9 screens provide an unbiased approach to establish the causal relationship between genotype and phenotype. By enabling genome-wide knockout of gene expression and subsequent analysis of resulting phenotypic changes, these screens have proven valuable [127, 128]. Additionally, the CRISPR system has been utilized for gene knockout and genetic mutation repair to facilitate in vitro disease modelling, offering significant time and labor savings compared to constructing animal models [129].

In recent years, CRISPR/Cas9 has emerged as a robust tool for investigating cartilage diseases. More recently, Chaudhry et al. [130] developed an efficient CRISPR/Cas9-mediated strategy for gene editing in primary human chondrocytes, enabling the investigation of miR-140-dependent mechanosensitive gene regulation. However, conventional 2D culture conditions still limit the assessment of intricate cellular functions and physiological characteristics of cartilage.

The integration of gene editing techniques with organoids has provided researchers with a powerful tool to investigate intrinsic developmental mechanisms through loss-of-function and gain-of-function studies conducted in vitro. Recently, CRISPR/Cas9 technology has been successfully applied to iPSC-based organoids, including neuronal, brain, intestinal, and colonic organoids. These advancements have significantly contributed to our improved understanding of human organogenesis, normal physiology, and disease pathology [131–134].

In a pioneering study by Ruiz et al. [135], CRISPR/Cas9 technique was employed on cartilaginous organoids to conduct functional characterization of the effects of OPG-XL in joint tissues. The researchers utilized hiPSCs derived from individuals within the affected CCAL1 family and employed CRISPR/Cas9 to repair hiPSCs, creating isogenic controls. These isogenic control cells were then utilized to establish in vitro organoid models of cartilage and bone, providing valuable insights into the effects of OPG-XL.

The combination of CRISPR/Cas9 and organoid technologies exhibits significant potential for advancing the study and treatment of genetic cartilaginous diseases, including Achondroplasia (ACH). ACH results from a mutation in the FGFR3 gene located on chromosome 4p16.31 [136]. Moreover, integrating CRISPR/Cas9 and organoid technologies can bridge the gap between potential therapeutic molecular mechanisms and their translation into treatments for human patients with OA and other cartilaginous diseases. Organoids, especially those derived from hiPSCs, serve as highly representative models of human diseases. By applying CRISPR-based mutagenesis to hiPSC-derived cartilaginous organoids, personalized therapy or precision medicine for cartilaginous diseases can be facilitated [137, 138].

Recent advancements in scRNA-seq technology, combined with CRISPR/Cas9 gene editing, have provided an unbiased approach to uncovering genotype–phenotype relationships at the single-cell level. For example, Dicks et al. [139] employed a CRISPR/Cas9-edited COL2A1-GFP knock-in reporter hiPSC line to identify a unique subpopulation of CPs with high chondrogenic potential. Subsequent analysis using scRNA-seq revealed distinct clusters within this population.

As highlighted earlier, the utilization of CRISPR/Cas9 gene editing technology in cartilaginous organoids has proven effective in elucidating human organogenesis, normal physiology, and disease pathology. Furthermore, implementing more sophisticated cartilaginous organoid culture systems can contribute to the evaluation of genome editing technology in terms of safety and efficiency.

However, CRISPR/Cas9 screening in organoids still faces certain limitations, primarily due to challenges associated with the manual handling of 3D organoids on a large scale. Improvements in sgRNA design, specifically tailored for organoids, can enhance phenotypic induction and penetrance, ultimately improving the CRISPR/Cas9 organoid screening platform to target patient-specific mutations or vulnerabilities. In due course, the emergence of microfluidic technology is expected to provide favorable conditions and timing for further advancements.

### Nourish every piece of land——microfluidic technology

Despite the significant progress achieved in the development of physiologically relevant cartilaginous organoids, several challenges persist. These challenges include heterogeneity, higher costs, and the need for high throughput in practical applications [140]. Addressing the limitations related to reproducibility and automation is crucial for the widespread utilization of cartilaginous organoids in clinical research. The integration of cartilaginous organoids with microfluidic systems, based on microphysiological technology, offers a promising avenue to overcome these technological challenges.

Microfluidic technology presents a powerful approach to establish complex biomolecule gradients that better mimic physiological conditions compared to conventional cell culture models. By replicating perfusion, mechanical forces, and other essential parameters for tissue and organ physiology, microfluidic systems enable a more comprehensive understanding of specific cellular responses and the fluidic and mechanical aspects of the cellular microenvironment [141]. Organ-on-a-chip devices, also known as organ chips, are microfluidic cell culture systems maintained under constant fluid flow. They serve as a means to bridge the gap between in vitro models and in vivo physiology [142].

The combination of organoid culture with microfluidic technologies offers several advantages [143]. First, microfluidic devices provide enhanced control over spontaneous morphogenesis, thus reducing variability. Second, automated operation reduces labor costs and minimizes human error. Third, miniaturized culture systems result in reduced reagent usage. Finally, microfluidic systems can expedite the maturation of organoids. Given the avascular, aneural state, and fibrillar composition of cartilage, microfluidic systems are particularly well suited for cartilaginous organoid studies. They enhance mass transfer, regulate cellular interactions, and allow for the adjustment of porosity.

A recent breakthrough by Rothbauer et al. [144] successfully established a microfluidic joint-on-a-chip organoid system. This system allows for the investigation of reciprocal cross-talk between individual synovial and cartilaginous organoids at the tissue level, providing a valuable model for studying arthritic diseases.

Microfluidic hydrogel-based scaffolds, fabricated using microfluidic devices, offer promising alternatives to traditional hydrogels used in cartilage tissue engineering [145]. The incorporation of microchannels enhances mass transfer, allowing for precise control over the distribution of chemical substances. This feature facilitates the creation of 3D structures that more accurately replicate native tissue, enabling the estimation of a particular tissue's functional performance [146, 147].

In the preceding discussion, we emphasize the capacity of cartilaginous organoids to simulate both physiological and pathological states of cartilage, including molecular mechanisms and signal transduction. However, despite the prevalent focus on chondrocyte pathobiology in most microphysiological models of OA, it is important to acknowledge that this disease affects the entire joint, including the influence of intra-articular pressure (IAP) and synovial fluid. Imbalanced mechanical stresses are identified as contributing factors to the pathogenesis of OA. Thus, mechanosensory activation during the onset and progression of OA represents a crucial yet often overlooked aspect of microsystems, demanding careful examination [148].

Moreover, systemic factors like age and sex, recognized as risk factors for OA, prove challenging to investigate using current joint-on-a-chip organoids.

Organ-on-a-chip platforms confront numerous practical considerations contingent on their intended application. Specifically, Joint-on-a-chip organoids are influenced by factors including device material selection, and the capacity for functional integration.

In the long term, the discovery of novel materials promises to foster technological advancements, potentially facilitating the large-scale fabrication of cartilaginous organoids integrated with microfluidic systems.

To enhance current disease models based on cartilaginous organoids, we propose the integration of anatomical and biomechanical considerations into next-generation microfluidic systems used in this context. The structure‒function relationship of an articular joint is highly intricate and multifaceted, involving diverse cellular, biochemical, and critical biophysical factors. Therefore, future models of OA that utilize cartilaginous organoids must enhance the control and precision of fluid-mechanical cues at the microscale.

To establish organoids as a reliable evaluation platform, it is essential to define specific technical standards and ensure the generation of functional units within a predetermined size range while maintaining consistent functionality. 3D bioprinting represents a novel approach that enables the creation of highly organized constructs.

### 3D bioprinting: set up the bed where organoids lay

3D bioprinting is an advanced technology that provides precise control over biophysical properties, such as organoid size, cell number, and structure, enabling the creation of tissue-like structures that closely resemble natural tissues [98].

While extensive discussions on the principles, classifications, characteristics, and applications of 3D bioprinting can be found in the literature, they surpass the scope of this paper [149, 150].

The application of 3D bioprinting to cartilaginous organoids is still in its early stages. However, recent advancements in fabrication techniques have made it feasible to utilize these methodologies for cartilaginous organoids using ADSCs and chondrocytes. Although hiPSCs hold immense potential for cartilaginous organoids and regenerative medicine, there are limited reports on bioprinting human 3D constructs based on hiPSCs. This is primarily due to the unique characteristics of hiPSCs, which present challenges for bioprinting. First, hiPSCs have low survival rates as single cells in culture, and dissociation into single cells is often a necessary step in most bioprinting procedures. Second, hiPSCs exhibit high responsiveness to environmental cues due to their embryonic-like nature and ability to respond to developmental signals. Last, hiPSCs tend to form clusters or colonies because of their epithelial character, which must be taken into account when employing nozzle-based bioprinting methods [151].

Cartilage tissue exhibits cellular heterogeneity and hierarchical organization across different zones of varying depth. However, accurately replicating zone-dependent characteristics in cartilaginous organoids, including size, ECM composition, and expression of anabolic and catabolic molecules, remains a challenge [152]. Traditionally, specific culture conditions are required to expand chondrocytes derived from different regions of the cartilage or stem cells undergoing distinct in vitro cartilage differentiation processes. Jonathan A [153] introduced a novel bioprinting-assisted tissue emergence (BATE) printing technology that utilizes stem cells and organoids as self-organizing building blocks. These blocks can be spatially arranged to form interconnected and evolving cellular structures. Through 3D bioprinting, individual cells or cellular aggregates that typically develop into randomly shaped small organoids can be induced to fuse and reorganize according to the imposed geometry and constraints. BATE printing technology holds promise for generating large-scale cartilaginous organoids with biological complexity.

Furthermore, Ludovicserex et al. [154] introduced a microfluidic-based print head that enables real-time adjustment of the print unit concentration, allowing for fibroblast bioprinting at concentrations of up to 10 million cells/mL. As the cell inoculation concentration plays a crucial role in 3D organoid culture, this method can yield more reliable and repeatable results.

Nonetheless, the technology remains in its experimental phase, signifying that various technical challenges must be addressed.

The generation of realistic cartilage incorporates diverse cell types of disparate shapes, thereby presenting a difficulty in determining the optimal printing parameters. Characteristically, 3D bioprinting exhibits slow printing speed and the attributes of the bio-ink, which include viscosity and cell density, are prone to alterations over time. This can notably influence the printing quality of cartilaginous organoids. Consequently, it is crucial to implement appropriate strategies to prevent ink desiccation during the printing process.

The hurdles that exist between laboratory research discoveries and commercial production are substantial. As it stands, 3D bioprinting within a laboratory context remains nascent and large-scale production of bio-products with high efficiency is currently unattainable. Hence, optimization of experimental apparatus and methodologies is a requisite for achieving rapid, and efficient large-scale biomanufacturing.

Although 3D bioprinting of cartilaginous organoids is still in its early stages, it offers immense potential. Future efforts should focus on improving cell viability postprinting, addressing limitations such as poor cytocompatibility and degradation-associated toxicity, and enhancing printing accuracy using existing technology. The integration of 3D bioprinting and microfluidic technologies may open new avenues for the development of cartilaginous organoids.

## Conclusion

**Fig. 6:**
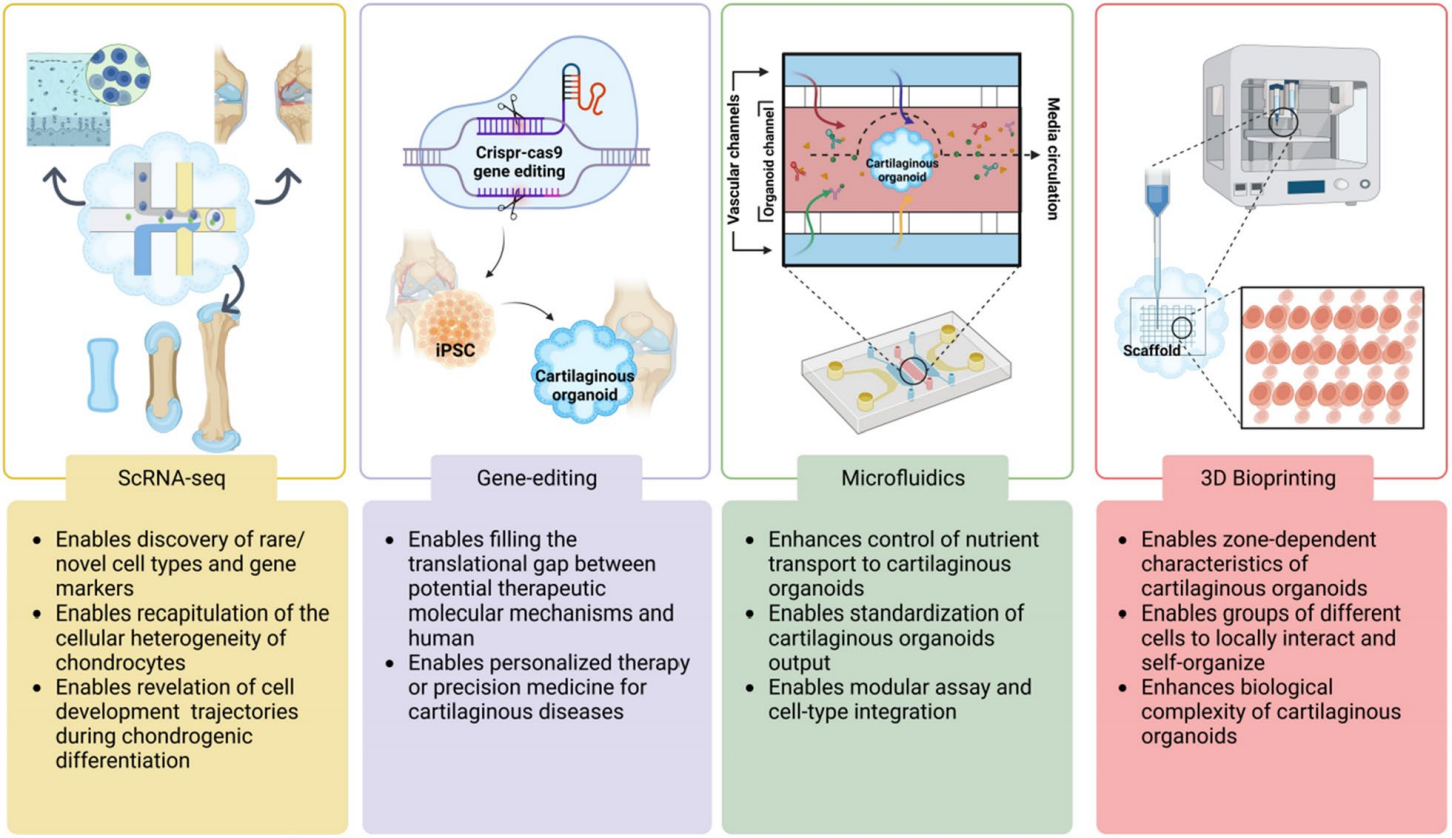
Perspectives of cartilaginous organoid technologies. Recent advances in scRNA-seq and CRISPR/Cas9 technologies could elucidate gene function on an unprecedented scale, which furthers the power for understanding human organogenesis, normal physiology, and disease pathology. Additionally, advancements in microfluidics and 3D bioprinting have enabled environmental control of nutrient mass transport and overall standardization of cartilaginous organoid size and spatial organization

Cartilaginous organoids serve as an ideal platform for large-scale mechanistic biology. They enable the establishment of a cartilaginous organoid cell atlas through high-throughput drug screening with molecular and phenotypic readouts, as well as single-cell multiomics analysis. The ongoing advancements in microfluidics, 3D bioprinting, and emerging nanomaterials offer a synergistic strategy to overcome limitations and leverage the advantages provided by cartilaginous organoids (Fig. 6). This approach allows for the construction of highly biocompatible microtissues at the centimeter scale and facilitates translation to the industry.

Collaborations among bioengineers, pharmacologists, clinicians, and developmental biologists, supported by cutting-edge technologies and multidisciplinary platforms, can accelerate the pace of discovery and enhance the precision of future clinical translations based on preclinical models of cartilaginous organoids.

## Data Availability

Not applicable.

## Abbreviations

3D: Three-dimensional
ASCs: Adult stem cells
PSCs: Pluripotent stem cells
2D: Two-dimensional
OA: Osteoarthritis
ESCs: Embryonic stem cells
iPSCs: Induced pluripotent stem cells
SCNT: Somatic cell nuclear transfer
CPs: Chondroprogenitors
SCs: Mesenchymal stem cells
PDCs: Periosteum-derived cells
hPSC: Human pluripotent stem cell
ECMs: Extracellular matrices
NCM: Notochordal cell-derived matrix
ADSCs: Adipose-derived stem cells
scRNA-seq: Single-cell sequencing
CPCs: Cartilage progenitor cells
VEGF: Vascular endothelial growth factor
cyiPSCs: Cynomolgus monkey induced pluripotent stem cells
cyiPS-Cart: CyiPSC-derived cartilaginous organoids
ACH: Achondroplasia
IAP: Intra-articular pressure
BATE: Bioprinting-assisted tissue emergence

## References

[1] Choudhury D, Ashok A, Naing MW (2020). Commercialization of organoids. Trends Mol Med.

[2] Rossi G, Manfrin A, Lutolf MP (2018). Progress and potential in organoid research. Nat Rev Genet.

[3] Smith E, Cochrane WJ (1946). Cystic organoid teratoma: (Report of a case). Can Med Assoc J.

[4] Sato T, Vries RG, Snippert HJ, van de Wetering M, Barker N, Stange DE, van Es JH, Abo A, Kujala P, Peters PJ, Clevers H (2009). Single Lgr5 stem cells build crypt-villus structures in vitro without a mesenchymal niche. Nature.

[5] Lancaster MA, Renner M, Martin CA, Wenzel D, Bicknell LS, Hurles ME, Homfray T, Penninger JM, Jackson AP, Knoblich JA (2013). Cerebral organoids model human brain development and microcephaly. Nature.

[6] Spence JR, Mayhew CN, Rankin SA, Kuhar MF, Vallance JE, Tolle K, Hoskins EE, Kalinichenko VV, Wells SI, Zorn AM (2011). Directed differentiation of human pluripotent stem cells into intestinal tissue in vitro. Nature.

[7] Wong AP, Bear CE, Chin S, Pasceri P, Thompson TO, Huan LJ, Ratjen F, Ellis J, Rossant J (2012). Directed differentiation of human pluripotent stem cells into mature airway epithelia expressing functional CFTR protein. Nat Biotechnol.

[8] Miller AJ, Dye BR, Ferrer-Torres D, Hill DR, Overeem AW, Shea LD, Spence JR (2019). Generation of lung organoids from human pluripotent stem cells in vitro 13. Nat Protoc.

[9] Wimmer RA, Leopoldi A, Aichinger M, Wick N, Hantusch B, Novatchkova M, Taubenschmid J, Hämmerle M, Esk C, Bagley JA (2019). Human blood vessel organoids as a model of diabetic vasculopathy. Nature.

[10] Karthaus WR, Iaquinta PJ, Drost J, Gracanin A, van Boxtel R, Wongvipat J, Dowling CM, Gao D, Begthel H, Sachs N (2014). Identification of multipotent luminal progenitor cells in human prostate organoid cultures. Cell.

[11] Takasato M, Er PX, Chiu HS, Maier B, Baillie GJ, Ferguson C, Parton RG, Wolvetang EJ, Roost MS, Lopes SM, Little MH (2016). Kidney organoids from human iPS cells contain multiple lineages and model human nephrogenesis. Nature.

[12] McCracken KW, Catá EM, Crawford CM, Sinagoga KL, Schumacher M, Rockich BE, Tsai YH, Mayhew CN, Spence JR, Zavros Y, Wells JM (2014). Modelling human development and disease in pluripotent stem-cell-derived gastric organoids. Nature.

[13] Cruz-Acuña R, Quirós M, Farkas AE, Dedhia PH, Huang S, Siuda D, García-Hernández V, Miller AJ, Spence JR, Nusrat A, García AJ (2017). Synthetic hydrogels for human intestinal organoid generation and colonic wound repair. Nat Cell Biol.

[14] Shirai H, Mandai M, Matsushita K, Kuwahara A, Yonemura S, Nakano T, Assawachananont J, Kimura T, Saito K, Terasaki H (2016). Transplantation of human embryonic stem cell-derived retinal tissue in two primate models of retinal degeneration. Proc Natl Acad Sci USA.

[15] Takebe T, Sekine K, Enomura M, Koike H, Kimura M, Ogaeri T, Zhang RR, Ueno Y, Zheng YW, Koike N (2013). Vascularized and functional human liver from an iPSC-derived organ bud transplant. Nature.

[16] Marti-Figueroa CR, Ashton RS (2017). The case for applying tissue engineering methodologies to instruct human organoid morphogenesis. Acta Biomater.

[17] Bilic J, Izpisua Belmonte JC (2012). Concise review: Induced pluripotent stem cells versus embryonic stem cells: close enough or yet too far apart?. Stem Cells.

[18] Garreta E, Kamm RD, de Chuva Sousa Lopes SM, Lancaster MA, Weiss R, Trepat X, Hyun I, Montserrat N (2021). Rethinking organoid technology through bioengineering. Nat Mater.

[19] Crispim JF, Ito K (2021). De novo neo-hyaline-cartilage from bovine organoids in viscoelastic hydrogels. Acta Biomater.

[20] Shieh AC, Athanasiou KA (2003). Principles of cell mechanics for cartilage tissue engineering. Ann Biomed Eng.

[21] Sanchez-Adams J, Leddy HA, McNulty AL, O'Conor CJ, Guilak F (2014). The mechanobiology of articular cartilage: bearing the burden of osteoarthritis. Curr Rheumatol Rep.

[22] Guilak F (2011). Biomechanical factors in osteoarthritis. Best Pract Res Clin Rheumatol.

[23] Xu J, Ji J, Jiao J, Zheng L, Hong Q, Tang H, Zhang S, Qu X, Yue B (2022). 3D Printing for bone-cartilage interface regeneration. Front Bioeng Biotechnol.

[24] Welton KL, Logterman S, Bartley JH, Vidal AF, McCarty EC (2018). Knee cartilage repair and restoration: common problems and solutions. Clin Sports Med.

[25] Dolzani P, Assirelli E, Pulsatelli L, Meliconi R, Mariani E, Neri S (2019). Ex vivo physiological compression of human osteoarthritis cartilage modulates cellular and matrix components. PLoS ONE.

[26] Sellam J, Berenbaum F (2013). Is osteoarthritis a metabolic disease? 22. Joint Bone Spine.

[27] Loeser RF, Goldring SR, Scanzello CR, Goldring MB (2012). Osteoarthritis: a disease of the joint as an organ. Arthritis Rheum.

[28] Giannoni P, Cancedda R (2006). Articular chondrocyte culturing for cell-based cartilage repair: needs and perspectives. Cells Tissues Organs.

[29] Yao Y, Wang Y (2013). ATDC5: an excellent in vitro model cell line for skeletal development. J Cell Biochem.

[30] Luo X, Wang J, Wei X, Wang S, Wang A (2020). Knockdown of lncRNA MFI2-AS1 inhibits lipopolysaccharide-induced osteoarthritis progression by miR-130a-3p/TCF4. Life Sci.

[31] Wolf F, Candrian C, Wendt D, Farhadi J, Heberer M, Martin I, Barbero A (2008). Cartilage tissue engineering using pre-aggregated human articular chondrocytes. Eur Cell Mater.

[32] Moreira Teixeira LS, Leijten JC, Sobral J, Jin R, van Apeldoorn AA, Feijen J, van Blitterswijk C, Dijkstra PJ, Karperien M (2012). High throughput generated micro-aggregates of chondrocytes stimulate cartilage formation in vitro and in vivo. Eur Cell Mater.

[33] Jeon JH, Yun BG, Lim MJ, Kim SJ, Lim MH, Lim JY, Park SH, Kim SW (2020). Rapid cartilage regeneration of spheroids composed of human nasal septum-derived chondrocyte in rat osteochondral defect model. Tissue Eng Regen Med.

[34] De Moor L, Beyls E, Declercq H (2020). Scaffold free microtissue formation for enhanced cartilage repair. Ann Biomed Eng.

[35] Lee JI, Sato M, Kim HW, Mochida J (2011). Transplantatation of scaffold-free spheroids composed of synovium-derived cells and chondrocytes for the treatment of cartilage defects of the knee. Eur Cell Mater.

[36] Wuelling M, Vortkamp A (2014). Cartilage explant cultures. Methods Mol Biol.

[37] Szponder T, Latalski M, Danielewicz A, Krać K, Kozera A, Drzewiecka B, Nguyen Ngoc D, Dobko D, Wessely-Szponder J (2022). Osteoarthritis: pathogenesis, animal models, and new regenerative therapies. J Clin Med.

[38] Zhang B, Radisic M (2017). Organ-on-a-chip devices advance to market. Lab Chip.

[39] Marx U, Akabane T, Andersson TB, Baker E, Beilmann M, Beken S, Brendler-Schwaab S, Cirit M, David R, Dehne EM (2020). Biology-inspired microphysiological systems to advance patient benefit and animal welfare in drug development. Altex.

[40] Panoutsopoulos AA (2021). Organoids, assembloids, and novel biotechnology: steps forward in developmental and disease-related neuroscience. Neuroscientist.

[41] Baptista LS, Kronemberger GS, Côrtes I, Charelli LE, Matsui RAM, Palhares TN, Sohier J, Rossi AM, Granjeiro JM (2018). Adult stem cells spheroids to optimize cell colonization in scaffolds for cartilage and bone tissue engineering. Int J Mol Sci.

[42] Adkar SS, Brunger JM, Willard VP, Wu CL, Gersbach CA, Guilak F (2017). Genome engineering for personalized arthritis therapeutics. Trends Mol Med.

[43] Umeda K, Zhao J, Simmons P, Stanley E, Elefanty A, Nakayama N (2012). Human chondrogenic paraxial mesoderm, directed specification and prospective isolation from pluripotent stem cells. Sci Rep.

[44] Adkar SS, Wu CL, Willard VP, Dicks A, Ettyreddy A, Steward N, Bhutani N, Gersbach CA, Guilak F (2019). Step-wise chondrogenesis of human induced pluripotent stem cells and purification via a reporter allele generated by CRISPR-Cas9 genome editing. Stem Cells.

[45] Dicks AR, Steward N, Guilak F, Wu CL (2023). Chondrogenic differentiation of human-induced pluripotent stem cells. Methods Mol Biol.

[46] Loh KM, Chen A, Koh PW, Deng TZ, Sinha R, Tsai JM, Barkal AA, Shen KY, Jain R, Morganti RM (2016). Mapping the pairwise choices leading from pluripotency to human bone, heart, and other mesoderm cell types. Cell.

[47] Craft AM, Rockel JS, Nartiss Y, Kandel RA, Alman BA, Keller GM (2015). Generation of articular chondrocytes from human pluripotent stem cells. Nat Biotechnol.

[48] O'Connor SK, Katz DB, Oswald SJ, Groneck L, Guilak F (2021). Formation of osteochondral organoids from murine induced pluripotent stem cells. Tissue Eng Part A.

[49] Tam WL, Freitas Mendes L, Chen X, Lesage R, Van Hoven I, Leysen E, Kerckhofs G, Bosmans K, Chai YC, Yamashita A (2021). Human pluripotent stem cell-derived cartilaginous organoids promote scaffold-free healing of critical size long bone defects. Stem Cell Res Ther.

[50] Hall GN, Tam WL, Andrikopoulos KS, Casas-Fraile L, Voyiatzis GA, Geris L, Luyten FP, Papantoniou I (2021). Patterned, organoid-based cartilaginous implants exhibit zone specific functionality forming osteochondral-like tissues in vivo. Biomaterials.

[51] Liu H, Yang L, Yu FF, Wang S, Wu C, Qu C, Lammi MJ, Guo X (2017). The potential of induced pluripotent stem cells as a tool to study skeletal dysplasias and cartilage-related pathologic conditions. Osteoarthritis Cartilage.

[52] Narsinh KH, Plews J, Wu JC (2011). Comparison of human induced pluripotent and embryonic stem cells: fraternal or identical twins?. Mol Ther.

[53] Hirschi KK, Li S, Roy K (2014). Induced pluripotent stem cells for regenerative medicine. Annu Rev Biomed Eng.

[54] Zhao J, Jiang WJ, Sun C, Hou CZ, Yang XM, Gao JG (2013). Induced pluripotent stem cells: origins, applications, and future perspectives. J Zhejiang Univ Sci B.

[55] Medvedev SP, Shevchenko AI, Zakian SM (2010). Induced pluripotent stem cells: problems and advantages when applying them in regenerative medicine. Acta Naturae.

[56] Liu Z, Tang Y, Lü S, Zhou J, Du Z, Duan C, Li Z, Wang C (2013). The tumourigenicity of iPS cells and their differentiated derivates. J Cell Mol Med.

[57] Puri MC, Nagy A (2012). Concise review: Embryonic stem cells versus induced pluripotent stem cells: the game is on. Stem Cells.

[58] Alford JW, Cole BJ (2005). Cartilage restoration, part 2: techniques, outcomes, and future directions. Am J Sports Med.

[59] Shimizu H, Yokoyama S, Asahara H (2007). Growth and differentiation of the developing limb bud from the perspective of chondrogenesis. Dev Growth Differ.

[60] Gao L, Orth P, Cucchiarini M, Madry H (2017). Effects of solid acellular type-I/III collagen biomaterials on in vitro and in vivo chondrogenesis of mesenchymal stem cells. Expert Rev Med Devices.

[61] Chamberlain G, Fox J, Ashton B, Middleton J (2007). Concise review: mesenchymal stem cells: their phenotype, differentiation capacity, immunological features, and potential for homing. Stem Cells.

[62] Dominici M, Le Blanc K, Mueller I, Slaper-Cortenbach I, Marini F, Krause D, Deans R, Keating A, Prockop D, Horwitz E (2006). Minimal criteria for defining multipotent mesenchymal stromal cells. The International society for cellular therapy position statement. Cytotherapy.

[63] Sakaguchi Y, Sekiya I, Yagishita K, Muneta T (2005). Comparison of human stem cells derived from various mesenchymal tissues: superiority of synovium as a cell source. Arthritis Rheum.

[64] Yoshimura H, Muneta T, Nimura A, Yokoyama A, Koga H, Sekiya I (2007). Comparison of rat mesenchymal stem cells derived from bone marrow, synovium, periosteum, adipose tissue, and muscle. Cell Tissue Res.

[65] Hamid AA, Idrus RB, Saim AB, Sathappan S, Chua KH (2012). Characterization of human adipose-derived stem cells and expression of chondrogenic genes during induction of cartilage differentiation. Clinics (Sao Paulo).

[66] Vishnubalaji R, Al-Nbaheen M, Kadalmani B, Aldahmash A, Ramesh T (2012). Comparative investigation of the differentiation capability of bone-marrow- and adipose-derived mesenchymal stem cells by qualitative and quantitative analysis. Cell Tissue Res.

[67] Baksh D, Yao R, Tuan RS (2007). Comparison of proliferative and multilineage differentiation potential of human mesenchymal stem cells derived from umbilical cord and bone marrow. Stem Cells.

[68] Vail DJ, Somoza RA, Caplan AI (2022). MicroRNA regulation of bone marrow mesenchymal stem cell chondrogenesis: toward articular cartilage. Tissue Eng Part A.

[69] Mareddy S, Crawford R, Brooke G, Xiao Y (2007). Clonal isolation and characterization of bone marrow stromal cells from patients with osteoarthritis. Tissue Eng.

[70] Pittenger MF, Mackay AM, Beck SC, Jaiswal RK, Douglas R, Mosca JD, Moorman MA, Simonetti DW, Craig S, Marshak DR (1999). Multilineage potential of adult human mesenchymal stem cells. Science.

[71] Larson BL, Yu SN, Park H, Estes BT, Moutos FT, Bloomquist CJ, Wu PB, Welter JF, Langer R, Guilak F, Freed LE (2019). Chondrogenic, hypertrophic, and osteochondral differentiation of human mesenchymal stem cells on three-dimensionally woven scaffolds. J Tissue Eng Regen Med.

[72] Diederichs S, Klampfleuthner FAM, Moradi B, Richter W (2019). Chondral differentiation of induced pluripotent stem cells without progression into the endochondral pathway. Front Cell Dev Biol.

[73] Nilsson Hall G, Mendes LF, Gklava C, Geris L, Luyten FP, Papantoniou I (2020). Developmentally engineered callus organoid bioassemblies exhibit predictive in vivo long bone healing. Adv Sci.

[74] Lin W, Wang M, Xu L, Tortorella M, Li G (2023). Cartilage organoids for cartilage development and cartilage-associated disease modeling. Front Cell Dev Biol.

[75] Martín AR, Patel JM, Zlotnick HM, Carey JL, Mauck RL (2019). Emerging therapies for cartilage regeneration in currently excluded 'red knee' populations. NPJ Regen Med.

[76] Ovsianikov A, Khademhosseini A, Mironov V (2018). The Synergy of scaffold-based and scaffold-free tissue engineering strategies. Trends Biotechnol.

[77] Mosaad EO, Chambers KF, Futrega K, Clements JA, Doran MR (2018). The Microwell-mesh: a high-throughput 3D prostate cancer spheroid and drug-testing platform. Sci Rep.

[78] Yamashita A, Morioka M, Yahara Y, Okada M, Kobayashi T, Kuriyama S, Matsuda S, Tsumaki N (2015). Generation of scaffoldless hyaline cartilaginous tissue from human iPSCs. Stem Cell Reports.

[79] Zhang H, Wu S, Chen W, Hu Y, Geng Z, Su J (2023). Bone/cartilage targeted hydrogel: strategies and applications. Bioact Mater.

[80] Vazin T, Schaffer DV (2010). Engineering strategies to emulate the stem cell niche. Trends Biotechnol.

[81] Allen JL, Cooke ME, Alliston T (2012). ECM stiffness primes the TGFβ pathway to promote chondrocyte differentiation. Mol Biol Cell.

[82] Orkin RW, Gehron P, McGoodwin EB, Martin GR, Valentine T, Swarm R (1977). A murine tumor producing a matrix of basement membrane. J Exp Med.

[83] Kaushik G, Ponnusamy MP, Batra SK (2018). Concise review: current status of three-dimensional organoids as preclinical models. Stem Cells.

[84] Schuh E, Hofmann S, Stok KS, Notbohm H, Müller R, Rotter N (2012). The influence of matrix elasticity on chondrocyte behavior in 3D. J Tissue Eng Regen Med.

[85] Bachmann B, Spitz S, Schädl B, Teuschl AH, Redl H, Nürnberger S, Ertl P (2020). Stiffness matters: fine-tuned hydrogel elasticity alters chondrogenic redifferentiation. Front Bioeng Biotechnol.

[86] Crispim JF, Ito K (2021). De novo neo-hyaline-cartilage from bovine organoids in viscoelastic hydrogels. Acta Biomater.

[87] Xiahou Z, She Y, Zhang J, Qin Y, Li G, Zhang L, Fang H, Zhang K, Chen C, Yin J (2020). Designer hydrogel with intelligently switchable stem-cell contact for incubating cartilaginous microtissues. ACS Appl Mater Interfaces.

[88] Mandrycky C, Wang Z, Kim K, Kim DH (2016). 3D bioprinting for engineering complex tissues. Biotechnol Adv.

[89] Vinatier C, Mrugala D, Jorgensen C, Guicheux J, Noël D (2009). Cartilage engineering: a crucial combination of cells, biomaterials and biofactors. Trends Biotechnol.

[90] Caliari SR, Harley BA (2014). Structural and biochemical modification of a collagen scaffold to selectively enhance MSC tenogenic, chondrogenic, and osteogenic differentiation. Adv Healthc Mater.

[91] Di Luca A, Szlazak K, Lorenzo-Moldero I, Ghebes CA, Lepedda A, Swieszkowski W, Van Blitterswijk C, Moroni L (2016). Influencing chondrogenic differentiation of human mesenchymal stromal cells in scaffolds displaying a structural gradient in pore size. Acta Biomater.

[92] Yang KC, Chen IH, Yang YT, Hsiao JK, Wang CC (2020). Effects of scaffold geometry on chondrogenic differentiation of adipose-derived stem cells. Mater Sci Eng C Mater Biol Appl.

[93] Kang H, Peng J, Lu S, Liu S, Zhang L, Huang J, Sui X, Zhao B, Wang A, Xu W (2014). In vivo cartilage repair using adipose-derived stem cell-loaded decellularized cartilage ECM scaffolds. J Tissue Eng Regen Med.

[94] Utomo L, Pleumeekers MM, Nimeskern L, Nürnberger S, Stok KS, Hildner F, van Osch GJ (2015). Preparation and characterization of a decellularized cartilage scaffold for ear cartilage reconstruction. Biomed Mater.

[95] Parmaksiz M, Dogan A, Odabas S, Elçin AE, Elçin YM (2016). Clinical applications of decellularized extracellular matrices for tissue engineering and regenerative medicine. Biomed Mater.

[96] Wu D, Wang X, Yang Y (2021). Chitosan-based high-mechanical double-network hydrogels: construction, modulation and applications. Acta Chim Sin.

[97] Sun Y, You Y, Jiang W, Wang B, Wu Q, Dai K (2020). 3D bioprinting dual-factor releasing and gradient-structured constructs ready to implant for anisotropic cartilage regeneration. Sci Adv.

[98] Bertassoni LE (2022). Bioprinting of complex multicellular organs with advanced functionality-recent progress and challenges ahead. Adv Mater.

[99] Li J, Dong S (2016). The signaling pathways involved in chondrocyte differentiation and hypertrophic differentiation. Stem Cells Int.

[100] Zhang L, Tang H, Xiahou Z, Zhang J, She Y, Zhang K, Hu X, Yin J, Chen C (2022). Solid multifunctional granular bioink for constructing chondroid basing on stem cell spheroids and chondrocytes. Biofabrication.

[101] Nakamura A, Murata D, Fujimoto R, Tamaki S, Nagata S, Ikeya M, Toguchida J, Nakayama K (2021). Bio-3D printing iPSC-derived human chondrocytes for articular cartilage regeneration. Biofabrication.

[102] Mendes LF, Tam WL, Chai YC, Geris L, Luyten FP, Roberts SJ (2016). Combinatorial analysis of growth factors reveals the contribution of bone morphogenetic proteins to chondrogenic differentiation of human periosteal cells. Tissue Eng Part C Methods.

[103] Matta C, Mobasheri A (2014). Regulation of chondrogenesis by protein kinase C: emerging new roles in calcium signalling. Cell Signal.

[104] Murphy MK, Huey DJ, Hu JC, Athanasiou KA (2015). TGF-β1, GDF-5, and BMP-2 stimulation induces chondrogenesis in expanded human articular chondrocytes and marrow-derived stromal cells. Stem Cells.

[105] Oldershaw RA, Baxter MA, Lowe ET, Bates N, Grady LM, Soncin F, Brison DR, Hardingham TE, Kimber SJ (2010). Directed differentiation of human embryonic stem cells toward chondrocytes. Nat Biotechnol.

[106] Boreström C, Simonsson S, Enochson L, Bigdeli N, Brantsing C, Ellerström C, Hyllner J, Lindahl A (2014). Footprint-free human induced pluripotent stem cells from articular cartilage with redifferentiation capacity: a first step toward a clinical-grade cell source. Stem Cells Transl Med.

[107] Wu CL, Dicks A, Steward N, Tang R, Katz DB, Choi YR, Guilak F (2021). Single cell transcriptomic analysis of human pluripotent stem cell chondrogenesis. Nat Commun.

[108] Cullier A, Cassé F, Manivong S, Contentin R, Legendre F, Garcia Ac A, Sirois P, Roullin G, Banquy X, Moldovan F (2022). Functionalized nanogels with endothelin-1 and bradykinin receptor antagonist peptides decrease inflammatory and cartilage degradation markers of osteoarthritis in a horse organoid model of cartilage. Int J Mol Sci.

[109] Zhou Z, Cui J, Wu S, Geng Z, Su J (2022). Silk fibroin-based biomaterials for cartilage/osteochondral repair. Theranostics.

[110] Donate R, Tamaddon M, Ribeiro V, Monzón M, Oliveira JM, Liu C (2022). Translation through collaboration: practice applied in BAMOS project in in vivo testing of innovative osteochondral scaffolds. Biomater Transl.

[111] Tamaddon M, Gilja H, Wang L, Oliveira JM, Sun X, Tan R, Liu C (2020). Osteochondral scaffolds for early treatment of cartilage defects in osteoarthritic joints: from bench to clinic. Biomater Transl.

[112] Han Y, Cao L, Li G, Zhou F, Bai L, Su J (2023). Harnessing nucleic acids nanotechnology for bone/cartilage regeneration. Small.

[113] Abe K, Yamashita A, Morioka M, Horike N, Takei Y, Koyamatsu S, Okita K, Matsuda S, Tsumaki N (2023). Engraftment of allogeneic iPS cell-derived cartilage organoid in a primate model of articular cartilage defect. Nat Commun.

[114] Thorup AS, Caxaria S, Thomas BL, Suleman Y, Nalesso G, Luyten FP, Dell'Accio F, Eldridge SE (2022). In vivo potency assay for the screening of bioactive molecules on cartilage formation. Lab Anim.

[115] Kleuskens MWA, Crispim JF, van Doeselaar M, van Donkelaar CC, Janssen RPA, Ito K (2023). Neo-cartilage formation using human nondegenerate versus osteoarthritic chondrocyte-derived cartilage organoids in a viscoelastic hydrogel. J Orthop Res.

[116] Abraham DM, Herman C, Witek L, Cronstein BN, Flores RL, Coelho PG (2022). Self-assembling human skeletal organoids for disease modeling and drug testing. J Biomed Mater Res B Appl Biomater.

[117] Grandi FC, Baskar R, Smeriglio P, Murkherjee S, Indelli PF, Amanatullah DF, Goodman S, Chu C, Bendall S, Bhutani N (2020). Single-cell mass cytometry reveals cross-talk between inflammation-dampening and inflammation-amplifying cells in osteoarthritic cartilage. Sci Adv.

[118] Lv Z, Han J, Li J, Guo H, Fei Y, Sun Z, Dong J, Wang M, Fan C, Li W (2022). Single cell RNA-seq analysis identifies ferroptotic chondrocyte cluster and reveals TRPV1 as an anti-ferroptotic target in osteoarthritis. EBioMedicine.

[119] Beumer J, Clevers H (2016). Regulation and plasticity of intestinal stem cells during homeostasis and regeneration. Development.

[120] Clevers H (2016). Modeling development and disease with organoids. Cell.

[121] Yin Y, Liu PY, Shi Y, Li P (2021). Single-cell sequencing and organoids: a powerful combination for modelling organ development and diseases. Rev Physiol Biochem Pharmacol.

[122] Tang Q, Iyer S, Lobbardi R, Moore JC, Chen H, Lareau C, Hebert C, Shaw ML, Neftel C, Suva ML (2017). Dissecting hematopoietic and renal cell heterogeneity in adult zebrafish at single-cell resolution using RNA sequencing. J Exp Med.

[123] Camp JG, Badsha F, Florio M, Kanton S, Gerber T, Wilsch-Bräuninger M, Lewitus E, Sykes A, Hevers W, Lancaster M (2015). Human cerebral organoids recapitulate gene expression programs of fetal neocortex development. Proc Natl Acad Sci USA.

[124] Czerniecki SM, Cruz NM, Harder JL, Menon R, Annis J, Otto EA, Gulieva RE, Islas LV, Kim YK, Tran LM (2018). High-throughput screening enhances kidney organoid differentiation from human pluripotent stem cells and enables automated multidimensional phenotyping. Cell Stem Cell.

[125] Hu H, Gehart H, Artegiani B, LÖpez-Iglesias C, Dekkers F, Basak O, van Esi J, de Chuva Sousa Lopes SM, Begthel H, Korving J (2018). Long-term expansion of functional mouse and human hepatocytes as 3D organoids. Cell.

[126] Smith C, Abalde-Atristain L, He C, Brodsky BR, Braunstein EM, Chaudhari P, Jang YY, Cheng L, Ye Z (2015). Efficient and allele-specific genome editing of disease loci in human iPSCs. Mol Ther.

[127] Cong L, Ran FA, Cox D, Lin S, Barretto R, Habib N, Hsu PD, Wu X, Jiang W, Marraffini LA, Zhang F (2013). Multiplex genome engineering using CRISPR/Cas systems. Science.

[128] Gilbert LA, Horlbeck MA, Adamson B, Villalta JE, Chen Y, Whitehead EH, Guimaraes C, Panning B, Ploegh HL, Bassik MC (2014). Genome-scale CRISPR-mediated control of gene repression and activation. Cell.

[129] Woo DH, Chen Q, Yang TL, Glineburg MR, Hoge C, Leu NA, Johnson FB, Lengner CJ (2016). Enhancing a Wnt-telomere feedback loop restores intestinal stem cell function in a human organotypic model of dyskeratosis congenita. Cell Stem Cell.

[130] Chaudhry N, Muhammad H, Seidl C, Downes D, Young DA, Hao Y, Zhu L, Vincent TL (2022). Highly efficient CRISPR-Cas9-mediated editing identifies novel mechanosensitive microRNA-140 targets in primary human articular chondrocytes. Osteoarthritis Cartilage.

[131] Blair JD, Hockemeyer D, Bateup HS (2018). Genetically engineered human cortical spheroid models of tuberous sclerosis. Nat Med.

[132] Fujii M, Matano M, Nanki K, Sato T (2015). Efficient genetic engineering of human intestinal organoids using electroporation. Nat Protoc.

[133] Kawasaki K, Fujii M, Sugimoto S, Ishikawa K, Matano M, Ohta Y, Toshimitsu K, Takahashi S, Hosoe N, Sekine S (2020). Chromosome engineering of human colon-derived organoids to develop a model of traditional serrated adenoma. Gastroenterology.

[134] Khan TA, Revah O, Gordon A, Yoon SJ, Krawisz AK, Goold C, Sun Y, Kim CH, Tian Y, Li MY (2020). Neuronal defects in a human cellular model of 22q11.2 deletion syndrome. Nat Med.

[135] Rodríguez Ruiz A, van Hoolwerff M, Sprangers S, Suchiman E, Schoenmaker T, Dibbets-Schneider P, Bloem JL, Nelissen R, Freund C, Mummery C (2022). Mutation in the CCAL1 locus accounts for bidirectional process of human subchondral bone turnover and cartilage mineralization. Rheumatology.

[136] Bonafe L, Cormier-Daire V, Hall C, Lachman R, Mortier G, Mundlos S, Nishimura G, Sangiorgi L, Savarirayan R, Sillence D (2015). Nosology and classification of genetic skeletal disorders 2015 revision. Am J Med Genet A.

[137] Nam SA, Seo E, Kim JW, Kim HW, Kim HL, Kim K, Kim TM, Ju JH, Gomez IG, Uchimura K (2019). Graft immaturity and safety concerns in transplanted human kidney organoids. Exp Mol Med.

[138] Quinn PM, Buck TM, Mulder AA, Ohonin C, Alves CH, Vos RM, Bialecka M, van Herwaarden T, van Dijk EHC, Talib M (2019). Human iPSC-derived retinas recapitulate the fetal CRB1 CRB2 Complex Formation And Demonstrate That Photoreceptors And Müller Glia Are Targets of AAV5. Stem Cell Reports.

[139] Dicks A, Wu CL, Steward N, Adkar SS, Gersbach CA, Guilak F (2020). Prospective isolation of chondroprogenitors from human iPSCs based on cell surface markers identified using a CRISPR-Cas9-generated reporter. Stem Cell Res Ther.

[140] Phipson B, Er PX, Combes AN, Forbes TA, Howden SE, Zappia L, Yen HJ, Lawlor KT, Hale LJ, Sun J (2019). Evaluation of variability in human kidney organoids. Nat Methods.

[141] Jang KJ, Otieno MA, Ronxhi J, Lim HK, Ewart L, Kodella KR, Petropolis DB, Kulkarni G, Rubins JE, Conegliano D (2019). Reproducing human and cross-species drug toxicities using a Liver-Chip. Sci Transl Med.

[142] Ingber DE (2022). Human organs-on-chips for disease modelling, drug development and personalized medicine. Nat Rev Genet.

[143] Baptista LS, Porrini C, Kronemberger GS, Kelly DJ, Perrault CM (2022). 3D organ-on-a-chip: The convergence of microphysiological systems and organoids. Front Cell Dev Biol.

[144] Rothbauer M, Byrne RA, Schobesberger S, Olmos Calvo I, Fischer A, Reihs EI, Spitz S, Bachmann B, Sevelda F, Holinka J (2021). Establishment of a human three-dimensional chip-based chondro-synovial coculture joint model for reciprocal cross talk studies in arthritis research. Lab Chip.

[145] Tolabi H, Davari N, Khajehmohammadi M, Malektaj H, Nazemi K, Vahedi S, Ghalandari B, Reis RL, Ghorbani F, Oliveira JM (2023). Progress of microfluidic hydrogel-based scaffolds and organ-on-chips for the cartilage tissue engineering. Adv Mater.

[146] Huang G, Wang S, He X, Zhang X, Lu TJ, Xu F (2013). Helical spring template fabrication of cell-laden microfluidic hydrogels for tissue engineering. Biotechnol Bioeng.

[147] Jiang W, Li M, Chen Z, Leong KW (2016). Cell-laden microfluidic microgels for tissue regeneration. Lab Chip.

[148] Statham P, Jones E, Jennings LM, Fermor HL (2022). Reproducing the biomechanical environment of the chondrocyte for cartilage tissue engineering. Tissue Eng Part B Rev.

[149] Ostrovidov S, Salehi S, Costantini M, Suthiwanich K, Ebrahimi M, Sadeghian RB, Fujie T, Shi X, Cannata S, Gargioli C (2019). 3D bioprinting in skeletal muscle tissue engineering. Small.

[150] Skardal A, Atala A (2015). Biomaterials for integration with 3-D bioprinting. Ann Biomed Eng.

[151] Gudapati H, Dey M, Ozbolat I (2016). A comprehensive review on droplet-based bioprinting: past, present and future. Biomaterials.

[152] Mizuno S, Takada E, Fukai N (2016). Spheroidal organoids reproduce characteristics of longitudinal depth zones in bovine articular cartilage. Cells Tissues Organs.

[153] Brassard JA, Nikolaev M, Hübscher T, Hofer M, Lutolf MP (2021). Recapitulating macro-scale tissue self-organization through organoid bioprinting. Nat Mater.

[154] Serex L, Sharma K, Rizov V, Bertsch A, McKinney JD, Renaud P (2021). Microfluidic-assisted bioprinting of tissues and organoids at high cell concentrations. Biofabrication.

